# Smoking cessation improves health status of patients with chronic diseases: evidence from a longitudinal study of older adults in China

**DOI:** 10.1186/s12889-025-22203-7

**Published:** 2025-03-11

**Authors:** Haoyu Zhu, Peng Xu, Yumeng Wei, Chuchen Zhao, Danni Zhao, Yaxin Li, Xiaobin Ma, Meng Wang, Huafeng Kang

**Affiliations:** The Comprehensive Breast Care Center, The Second Affiliated Hospital of Xi’an Jiaotong University, Xi’an, Shaanxi China

**Keywords:** Smoking cessation, Health status, CHARLS, Older adults

## Abstract

**Background:**

Smoking is a well-documented risk factor for numerous chronic diseases, and cessation is correlated with enhanced health outcomes. Nonetheless, the precise effects of smoking cessation on the health status of older adults with chronic conditions in China have not been thoroughly quantified.

**Objective:**

This study aims to quantitatively assess the correlations between smoking cessation and enhancements in the health outcomes of elderly Chinese individuals with chronic diseases.

**Method:**

This research drew upon data from the China Health and Retirement Longitudinal Study (CHARLS). A cohort of 9914 participants was ultimately included in our analysis. Group comparisons and linear regression analyses were utilized. The investigation delved into health status scores, hematological markers, and physiological parameters.

**Result:**

With each additional year of smoking cessation, former smokers demonstrated improved self-rated health and reduced EQ-5D-3L scores. Regression analysis unveiled a positive correlation between smoking cessation and enhanced self-assessed health (β estimate = 0.198), while a notable adverse effect was observed in EQ-5D-3L scores (β estimate = -0.179) and grip strength (β estimate = -2.530). Blood biomarkers also displayed noteworthy relationships with smoking cessation, showcasing rehabilitation in LDL cholesterol, total cholesterol, glucose, cystatin C, creatinine, HbA1c, and uric acid levels.

**Conclusion:**

This research provides evidence highlighting the favorable health ramifications associated with smoking cessation in elderly individuals with chronic illnesses. Noteworthy improvements in both subjective health assessments and blood-based markers were observed post-smoking cessation, with benefits becoming more prominent with prolonged abstinence. These results underscore the vital importance of smoking cessation in the holistic care of chronic conditions and broader health enhancement endeavors. Further validation of these findings through an extended follow-up period is anticipated to bolster these conclusions with increased confidence.

**Supplementary Information:**

The online version contains supplementary material available at 10.1186/s12889-025-22203-7.

## Introduction

Smoking is widely acknowledged as a primary behavioral risk factor in global public health. Over the past seven decades, a plethora of studies [1–4] has elucidated the connections between smoking and a range of chronic conditions, including but not limited to cancer [5], hypertension [6, 7], stroke [8], asthma [9], and myocardial infarction [10]. Undeniably, tobacco smoking emerges as a crucial determinant of health in individuals with chronic diseases. Recent research has underscored the possibility for smokers to prolong their life expectancy by up to a decade through smoking cessation [11]. Moreover, smoking cessation has been recognized as a fundamental element in the management of chronic diseases [12–14]. Wang Z et al [15] screened 13,460 literatures and included 11 studies of chronic obstructive pulmonary disease (COPD) patients. Using systematic review and meta-analysis, they analyzed forced expiratory volume in one second percentage predicted (FEV1% predicted), FEV1/forced vital capacity (FVC), 6-min walk test (6-MWT), and etc. They concluded that smoking cessation is beneficial to COPD patients, which can improve lung function, alleviate symptoms, enhance exercise tolerance, and may reduce mortality. Pan B et al [16] searched databases and included 14 studies with 303,134 subjects. Through meta-analysis of smoking and stroke factors like smoking status and gender, they found smoking increases stroke risk and cessation helps. Other reviews and meta-analyses have also reflected the benefits of smoking cessation on chronic patients, such as asthma [17], and ischemic heart disease [18].

Notably, China leads the world in tobacco consumption, with Chinese men representing approximately 40% of global cigarette usage [19]. Disturbing trends in cigarette consumption among Chinese youth over the last two decades indicate a potential rise in mortality risks linked to tobacco use for men in urban and rural regions alike [20]. As reported in other research [21], the smoking prevalence of the adults over 45 years old in a province of China is over 20%, much more higher than that in Europe and the United States of America.

Consequently, this study aims to quantify the correlations between smoking cessation and enhancements in the health status of elderly Chinese individuals with chronic conditions. All data utilized in this research were sourced from the China Health and Retirement Longitudinal Study (CHARLS) database. The most recent update of the CHARLS database in November 2023 enables us to access the most up-to-date information from China. The analysis encompassed health status assessments, blood biomarkers, and physical measurements.

## Methods

### Study design and sample source

The CHARLS represents a comprehensive initiative aimed at gathering high-quality microdata concerning Chinese households and individuals aged 45 and older. The inaugural national survey of CHARLS took place in 2011, encompassing 450 villages, 150 countries, and 28 provinces, involving approximately 17,000 individuals from 10,000 households. This survey captures fundamental demographic details about the participants and their families, intra-family financial transfers, the health status of respondents, healthcare access and insurance coverage, employment particulars, income, expenditures, assets, and more. Moreover, CHARLS involved 13 physical assessments and the collection of blood samples. To date, CHARLS has released five waves of data: the national baseline survey (Wave 1, 2011), and subsequent follow-up surveys (Wave 2, 2013; Wave 3, 2015; Wave 4, 2018; Wave 5, 2020). The CHARLS datasets are accessible for download on the CHARLS homepage at http://charls.pku.edu.cn/en. Approval for the CHARLS survey project was obtained from the Biomedical Ethics Committee of Peking University, and all participants provided informed consent [22].

In this study, we selected data from all five waves of the CHARLS database. The inclusion criteria were as follows: (1) individuals diagnosed with any chronic disease in Wave 1; (2) individuals who underwent treatment for chronic diseases. To avoid bias stemming from treatment for chronic diseases and to ascertain the true impact of smoking cessation, we only selected individuals who received treatment. These chronic diseases encompassed hypertension, diabetes, cancer, chronic lung disease, heart attack, stroke, psychiatric problems, arthritis or rheumatism, dyslipidemia, any liver disease, any kidney disease, any stomach or other digestive disease, and asthma. Based on previous studies [5–10], hypertension, asthma, cancer, chronic lung disease, heart attack, and stroke were considered to be related to smoking. The exclusion criteria were as follows: (1) individuals aged under 45 years in Wave 1; (2) individuals quitting smoking outside the follow-up period. We used the STROBE cohort checklist when writing our report [23].

### Variables

To demonstrate the association between smoking cessation and chronic disease status, the following variables were selected for analysis: (1) demographic factors including age, gender, marital status, educational background, public insurance, and residence area; (2) smoking status and duration since quitting; (3) chronic disease status and specific disease types. These participants were initially classified into three main groups based on their smoking status: non-smokers, current smokers, and former smokers. According some similar researches [24, 25], former smokers were defined as individuals who previously smoked but have since discontinued this habit.

To assess health status, we utilized various metrics, including self-rating, blood analyses, physical measurements, and the EuroQoL 5-Dimension 3-Level (EQ-5D-3L) questionnaire. In CHARLS, participants were requested to rate their health on a scale ranging from 1 to 5, with 1 indicating the worst and 5 indicating the best health. Blood analyses were conducted in Wave 1 and Wave 3, while physical measurements were conducted in Wave 1, Wave 2, and Wave 3 [26], although not all respondents underwent these assessments. The blood analyses encompassed parameters such as hemoglobin, hematocrit, white blood cell count (WBC), platelet count, mean corpuscular volume (MCV), C-reactive protein (CRP), glycosylated hemoglobin, type A1c (HbA1c), total cholesterol, high-density lipoprotein (HDL) cholesterol, low-density lipoprotein (LDL) cholesterol, triglycerides, glucose, blood urea nitrogen (BUN), creatinine, uric acid, and cystatin C. Physical measurements included walk measurements, blood pressure measurements, hand grip strength measurements, body mass index (BMI), lung function measurements, balance tests, and chair stand tests. Detailed units for these variables can be found in Table S-1.

The EQ-5D-3L questionnaire was employed to assess the health-related quality of life (HRQOL) of our participants. HRQOL was evaluated across five dimensions (5D): mobility (MO), self-care (SC), usual activities (UA), pain/discomfort (PD), and anxiety/depression (AD). Each dimension comprised three levels (3L): no problem, moderate problems, and severe problems [27]. Previous research has successfully developed Chinese utility values for EQ-5D-3L health states using the time trade-off method and established a scale and formula specific to Chinese residents [28–30](Table S-2). Based on other researchs [6], we selected five parts from CHARLS for each dimension: MO: Difficulty with Shopping for Groceries, SC: Difficulty with Preparing Hot Meals, UA: Difficulty with Household Chores, PD: Troubled with Body Pain, AD: Center for Epidemiologic Studies Depression Scale-10 (CESD-10) questionnaire in CHARLS, recognized as an effective mental health assessment tool for the elderly [31, 32].

### Data analysis

Descriptive analyses of demographics and health status were conducted using frequency counts, proportionate ratios, means, and standard deviations. Group comparisons for continuous variables were performed using the Wilcoxon rank-sum test. For former smokers, the parameters before and after the cessation were calculated. For example, if a respondent quit smoking in 2014, the record in Wave 3 (2015), Wave 4 (2018), and Wave 5 (2020) would be described as 1-year cessation, 3-year cessation, and 6-year cessation respectively. The average of Wave 1 and Wave 2 was considered a pre-cessation value, and the average of the other three waves was considered a post-cessation value. All blood analysis outcomes were natural log-transformed to improve normality, and thus, results were presented as percentage differences: (exp (β-coefficient) -1) × 100%. To investigate the relationships between smoking status and the parameters included in the study, univariable and multivariable linear regression analyses were employed. Model 1 (M1) featured a binary indicator for smoking cessation status, while Model 2 (M2) adjusted for demographic variables. Moreover, the duration since quitting smoking was taken into account. The statistical analyses were executed using R (version 4.3.3) with a significance level of α = 0.05. Most of the results were rounded up to two decimals places.

## Result

### Descriptive statistics

**Fig. 1:**
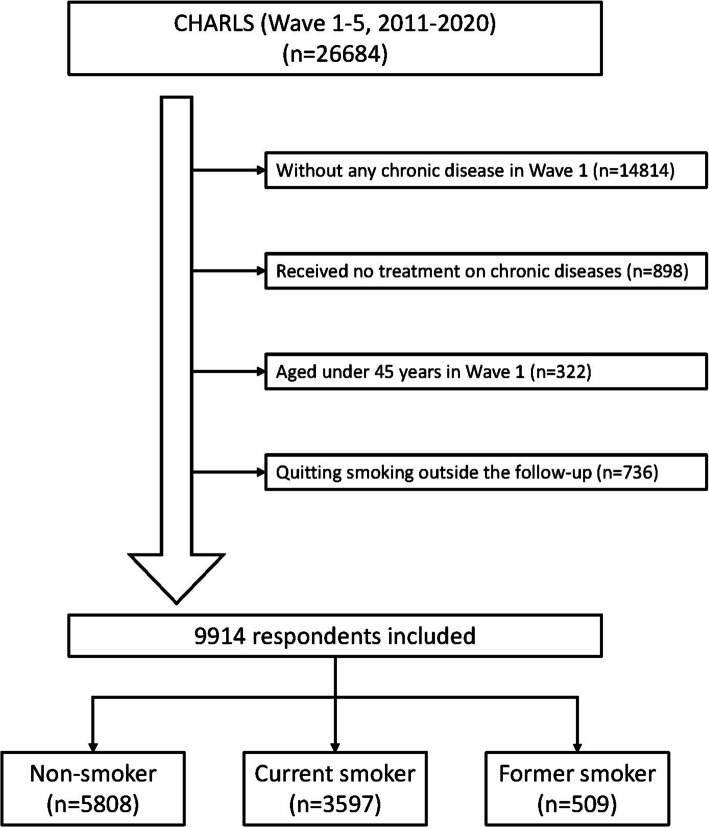
Flowchart for inclusion and exclusion in our study

Finally, a cohort of 9,914 individuals was identified for subsequent investigations (Fig. 1). Table 1 details the descriptive analysis of demographic characteristics. The mean age of the former group was slightly higher than the overall. Around 80% of male participants enrolled in the study had a history of smoking, aligning with findings from prior research studies.

**Table 1:** Characteristics of included respondents in the study

Variables	Total	Current smoker	Former smoker	Non-smoker	*P* value
	*N* = 9914	*N* = 3597	*N* = 509	*N* = 5808	
Age (Mean (SD))	60.62 (9.77)	61.13 (9.82)	64.53 (8.48)	59.95 (9.74)	< 0.001
Gender (%)					< 0.001
Male	4299 (43.36%)	3069 (85.32%)	455 (89.39%)	775 (13.34%)	
Female	5615 (56.64%)	528 (14.68%)	54 (10.61%)	5033 (86.66%)	
Marital status^a^ (%)					< 0.001
Partnered	9142 (92.22%)	3272 (90.96%)	509 (100.00%)	5361 (92.32%)	
Non-partnered	771 (7.78%)	325 (9.04%)	0 (0.00%)	446 (7.68%)	
Education (%)					< 0.001
Lower secondary or lower	8845 (89.24%)	3165 (87.99%)	438 (86.05%)	5242 (90.30%)	
Upper secondary or higher	1066 (10.76%)	432 (12.01%)	71 (13.95%)	563 (9.70%)	
Residence (%)					0.019
Urban	3995 (40.30%)	1426 (39.64%)	179 (35.17%)	2390 (41.15%)	
Rural	5919 (59.70%)	2171 (60.36%)	330 (64.83%)	3418 (58.85%)	
Drinking alcohol (%)					< 0.001
Yes	5496 (55.44%)	2816 (78.29%)	428 (84.09%)	2252 (38.77%)	
No	4418 (44.56%)	781 (21.71%)	81 (15.91%)	3556 (61.23%)	
With public insurance (%)					0.163
Yes	9388 (94.79%)	3391 (94.33%)	489 (96.07%)	5508 (94.97%)	
No	516 (5.21%)	204 (5.67%)	20 (3.93%)	292 (5.03%)	
With smoke related disease (%)					0.012
Yes	5965 (60.17%)	2233 (62.08%)	306 (60.12%)	3426 (58.99%)	
No	3949 (39.83%)	1364 (37.92%)	203 (39.88%)	2382 (41.01%)	

^a^Those who married but not living with a spouse, separated, divorced, widowed, and never married were considered non-partnered

Table 2 presented the distribution of respondents with various chronic diseases, revealing that hypertension and arthritis or rheumatism emerged as the predominant chronic conditions among the participants. Each chronic ailment displayed a relatively consistent prevalence across all groups. However, concerning respiratory system disorders, the prevalence rate among current smokers was approximately double that observed among non-smokers.

**Table 2:** Number of respondents with each type of chronic disease

	Overall	Never	Current	Former
	*N* = 9914	*N* = 5808	*N* = 3597	*N* = 509
**Smoke related disease**				
Hypertension	3983 (40.18%)	2405 (41.41%)	1386 (38.53%)	192 (37.72%)
Cancer or a malignant tumor	139 (1.40%)	85 (1.46%)	48 (1.33%)	6 (1.18%)
Lung disease	1420 (14.32%)	659 (11.35%)	680 (18.90%)	81 (15.91%)
Asthma	645 (6.51%)	287 (4.94%)	321 (8.92%)	37 (7.27%)
Heart problems	1890 (19.06%)	1137 (19.58%)	659 (18.32%)	94 (18.47%)
Stroke	417 (4.21%)	218 (3.75%)	183 (5.09%)	16 (3.14%)
**Non-smoke related disease**				
Psychiatric problems	212 (2.14%)	109 (1.88%)	93 (2.59%)	10 (1.96%)
Arthritis or rheumatism	4895 (49.37%)	3037 (52.29%)	1627 (45.23%)	231 (45.38%)
Dyslipidemia	1428 (14.40%)	889 (15.31%)	460 (12.79%)	79 (15.52%)
Liver disease	507 (5.11%)	259 (4.46%)	219 (6.09%)	29 (5.70%)
Kidney disease	830 (8.37%)	439 (7.56%)	349 (9.70%)	42 (8.25%)
Digestive disease	3229 (32.57%)	1940 (33.40%)	1142 (31.75%)	147 (28.88%)
Diabetes or high blood sugar	937 (9.45%)	582 (10.02%)	318 (8.84%)	37 (7.27%)

One respondent may be diagnosed with more than one type of chronic disease

*Never* Non-smokers, *Current* Current smokers, *Former* Quitters

### Increasing self-rate and decreasing EQ-5D-3L scores

**Fig. 2:**
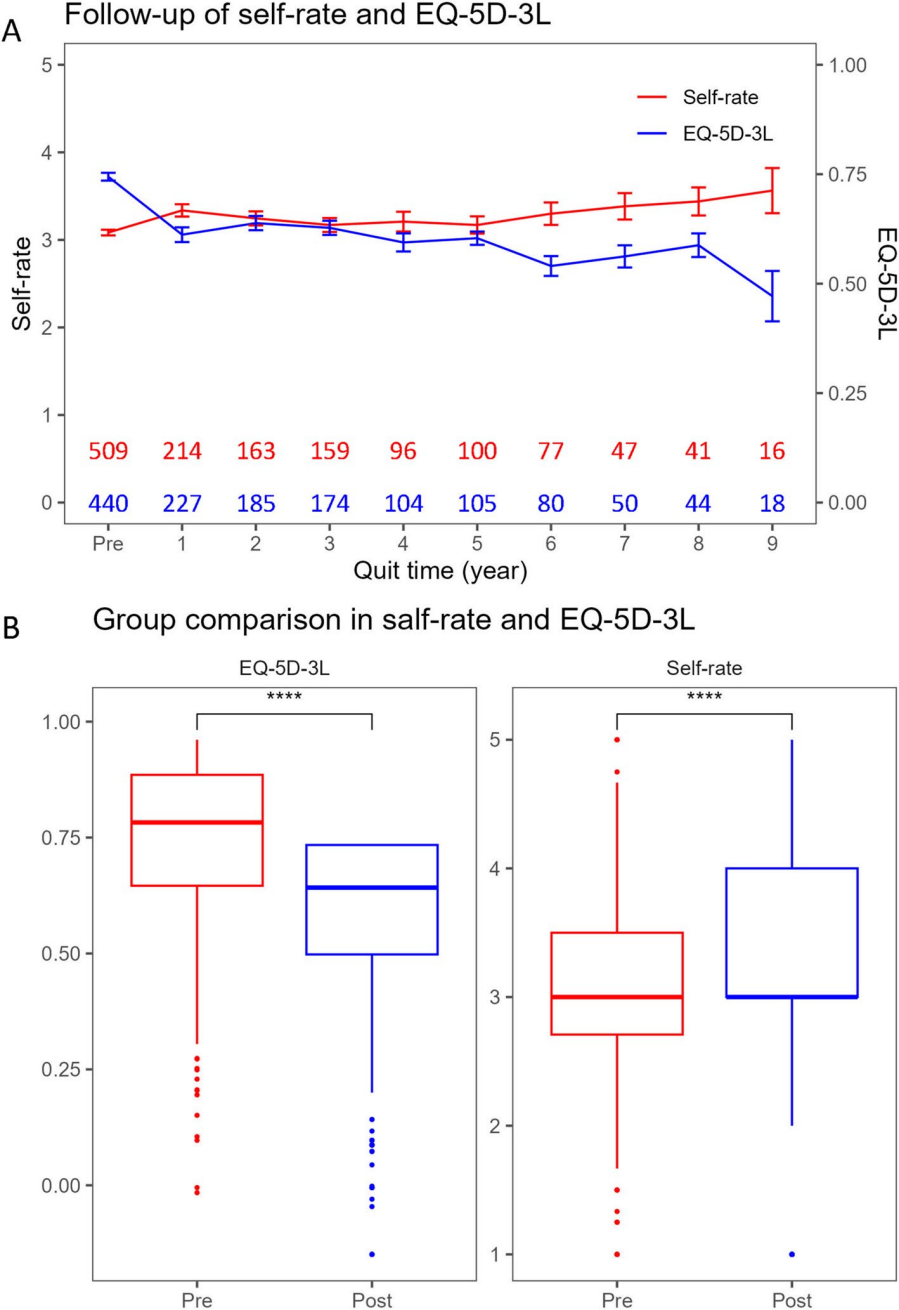
**A** The trend of self-rate and EQ-5D-3L scores for former smokers. The red and blue numbers indicate the number of respondents for each corresponding quitting year. **B** Self-rate and EQ-5D-3L scores for former smokers before and after cessation. *: *p* < 0.05, **: *p* < 0.01, ***: *p* < 0.001, ****: *p* < 0.0001

Figure 2A showed the trend of self-rate and EQ-5D-3L scores among former smokers. Upon analysis, we observed a progressive rise in self-rating scores over time, contrasting with a declining trend in EQ-5D-3L scores. Figure 2B illustrates a comparison between pre- and post-cessation periods. It revealed that individuals who quit smoking exhibited higher self-rating scores but lower EQ-5D-3L scores. Therefore, a detailed examination of EQ-5D-3L data indicated that, in comparison to smokers, quitters demonstrated an increase in the AD coefficient by 0.117, along with incremental changes in the SC, UA, and PD coefficients by 0.017, 0.018, and 0.015, respectively. This analysis suggested that the reduction in EQ-5D-3L scores primarily stemmed from the elevated AD coefficient (Table S-3).

### Health status comparisons between all subgroups

As shown in Table 3, almost all parameters in the current smoker and pre-cessation groups displayed similar levels, except for self-rate, EQ-5D-3L, HbA1c, grip strength, and lung function. In blood analyses, compared with the pre-cessation group, the post-cessation group demonstrated a decline in hemoglobin, total cholesterol, LDL cholesterol, and cystatin C, alongside an increase in creatinine, HbA1c, and uric acid. Non-smokers exhibited lower values in hemoglobin, hematocrit, MCV, BUN, creatinine, uric acid, cystatin C, limb strength, and lung function, while a higher level in HbA1c compared to the pre-cessation group.

**Table 3:** Group comparisons between different smoking status

Variables	Total	Pre (Ref)	Post	Current	Never	*P* value (overall)
Self-rate	*N* = 10,825	*N* = 509	*N*** = 913**	*N*** = 3597**	*N*** = 5806**	< 0.001
	3.25 (0.74)	3.08 (0.73)	**3.27 (1.03)**	**3.23 (0.72)**	**3.28 (0.70)**	
EQ-5D-3L	*N* = 10,331	*N* = 440	*N*** = 987**	*N*** = 3361**	*N*** = 5543**	< 0.001
	0.62 (0.21)	0.74 (0.19)	**0.58 (0.25)**	**0.64 (0.22)**	**0.61 (0.20)**	
**Blood analyses**						
WBC	*N* = 7796	*N* = 85	*N* = 158	*N* = 2814	*N* = 4739	< 0.001
	6.16 (1.75)	6.32 (2.12)	6.17 (1.82)	6.34 (1.82)	6.05 (1.69)	
Hemoglobin	*N* = 7795	*N* = 85	*N*** = 158**	*N* = 2814	*N*** = 4738**	< 0.001
	14.0 (1.96)	15.0 (2.16)	**14.3 (1.53)**	14.8 (1.99)	**13.5 (1.78)**	
Hematocrit	*N* = 7902	*N* = 86	*N* = 158	*N* = 2849	*N*** = 4809**	< 0.001
	41.2 (5.40)	43.5 (5.86)	43.1 (4.78)	43.0 (5.38)	**40.0 (5.07)**	
MCV	*N* = 7795	*N* = 85	*N* = 158	*N* = 2813	*N*** = 4739**	< 0.001
	90.8 (7.74)	92.0 (7.29)	91.7 (7.10)	92.2 (8.03)	**89.9 (7.45)**	
Platelet	*N* = 7793	*N* = 85	*N* = 158	*N* = 2812	*N* = 4738	< 0.001
	208 (69.5)	206 (67.9)	213 (90.4)	202 (69.1)	212 (68.7)	
BUN	*N* = 7881	*N* = 84	*N* = 160	*N* = 2835	*N*** = 4802**	< 0.001
	15.8 (4.55)	17.2 (5.74)	16.8 (5.64)	16.4 (4.49)	**15.5 (4.48)**	
Creatinine	*N* = 7873	*N* = 84	*N*** = 160**	*N* = 2832	*N*** = 4797**	< 0.001
	0.80 (0.31)	0.89 (0.33)	**0.99 (0.85)**	0.88 (0.30)	**0.75 (0.26)**	
Total cholesterol	*N* = 7880	*N* = 84	*N*** = 160**	*N* = 2835	*N* = 4801	< 0.001
	191 (36.7)	190 (36.5)	**177 (32.4)**	185 (36.5)	195 (36.5)	
Triglycerides	*N* = 7880	*N* = 84	*N* = 160	*N* = 2835	*N* = 4801	0.005
	143 (97.7)	153 (181)	147 (84.9)	138 (107)	146 (89.9)	
HDL cholesterol	*N* = 7882	*N* = 84	*N* = 160	*N* = 2837	*N* = 4801	0.004
	50.9 (13.2)	49.9 (18.2)	48.2 (11.0)	50.5 (14.3)	51.3 (12.5)	
LDL cholesterol	*N* = 7881	*N* = 84	*N*** = 160**	*N* = 2835	*N* = 4802	< 0.001
	111 (31.7)	112 (35.0)	**97.5 (24.6)**	107 (31.1)	114 (31.8)	
CRP	*N* = 7883	*N* = 84	*N* = 160	*N* = 2836	*N* = 4803	0.003
	3.14 (6.98)	3.69 (8.92)	3.28 (6.96)	3.51 (7.90)	2.91 (6.33)	
Glucose	*N* = 7875	*N* = 84	*N* = 160	*N* = 2834	*N* = 4797	0.021
	110 (38.1)	111 (34.3)	101 (19.3)	110 (38.1)	110 (38.7)	
HbA1c	*N* = 7885	*N* = 86	*N*** = 160**	*N*** = 2835**	*N*** = 4804**	< 0.001
	5.66 (0.99)	5.25 (0.77)	**5.92 (0.68)**	**5.61 (0.96)**	**5.69 (1.02)**	
Uric acid	*N* = 7887	*N* = 84	*N*** = 160**	*N* = 2838	*N*** = 4805**	< 0.001
	4.70 (1.29)	4.98 (1.40)	**5.72 (1.47)**	5.07 (1.33)	**4.44 (1.19)**	
Cystatin C	*N* = 7771	*N* = 74	*N*** = 54**	*N* = 2838	*N*** = 4805**	< 0.001
	4.69 (1.29)	5.82 (1.49)	**5.79 (1.49)**	5.07 (1.33)	**4.44 (1.19)**	
**Physical measures**						
Walk time	*N* = 5628	*N* = 39	*N* = 152	*N* = 2117	*N* = 3320	< 0.001
	4.35 (2.34)	4.34 (1.67)	3.47 (2.27)	4.31 (2.42)	4.42 (2.29)	
Systolic pressure	*N* = 9068	*N* = 92	*N* = 232	*N* = 3335	*N* = 5409	0.218
	132 (20.0)	130 (18.0)	131 (17.1)	133 (20.2)	132 (20.0)	
Diastolic pressure	*N* = 9068	*N* = 92	*N* = 232	*N* = 3335	*N* = 5409	< 0.001
	76.6 (11.4)	77.8 (11.1)	79.4 (11.4)	77.3 (11.6)	76.1 (11.2)	
Pulse	*N* = 9067	*N* = 92	*N* = 232	*N* = 3334	*N* = 5409	0.110
	73.8 (9.68)	74.1 (12.5)	73.9 (12.9)	74.1 (9.98)	73.6 (9.27)	
Grip strength	*N* = 8995	*N* = 90	*N* = 231	*N*** = 3309**	*N*** = 5365**	< 0.001
	29.1 (9.84)	37.6 (9.95)	36.0 (10.2)	**33.8 (10.1)**	**25.7 (8.07)**	
BMI	*N* = 8976	*N* = 91	*N* = 234	*N* = 3302	*N* = 5349	< 0.001
	24.3 (14.9)	23.4 (3.70)	23.7 (3.81)	23.5 (7.14)	24.9 (18.5)	
Lung function	*N* = 8947	*N* = 88	*N* = 230	*N*** = 3292**	*N*** = 5337**	< 0.001
	282 (114)	344 (140)	333 (135)	**311 (129)**	**260 (96.4)**	
Balance	*N* = 8642	*N* = 89	*N* = 223	*N* = 3165	*N* = 5165	< 0.001
	3.74 (0.39)	3.79 (0.40)	3.83 (0.38)	3.79 (0.36)	3.71 (0.40)	
Chair 5 s	*N* = 8648	*N* = 86	*N* = 225	*N* = 3170	*N* = 5167	< 0.001
	11.0 (4.20)	10.1 (3.01)	9.62 (3.24)	10.8 (4.14)	11.2 (4.26)	
Chair 5 num	*N* = 8667	*N* = 86	*N* = 225	*N* = 3180	*N* = 5176	0.056
	4.99 (0.15)	5.00 (0.00)	5.00 (0.00)	4.99 (0.19)	4.99 (0.11)	

All significate outcomes compared with pre-cessation group (*P* < 0.05) were bold in the table

Each project group's participant count, mean, and standard deviation of the indicators are described, with units indicated in Table S-1

*Current* Current smokers, *Pre* Before cessation, *Post* After cessation, *Never* Non-smokers

*Abbreviation*: *EQ-5D-3L* EuroQoL 5-Dimension 3-Level, *WBC* white blood cell count, *MCV* platelet count, mean corpuscular volume, *BUN* blood urea nitrogen, *HDL* high-density lipoprotein, *LDL* low-density lipoprotein, *CRP* C-reactive protein, *HbA1c* glycosylated hemoglobin, type A1c, *BMI* body mass index

### Regression analysis revealed health benefits

**Fig. 3:**
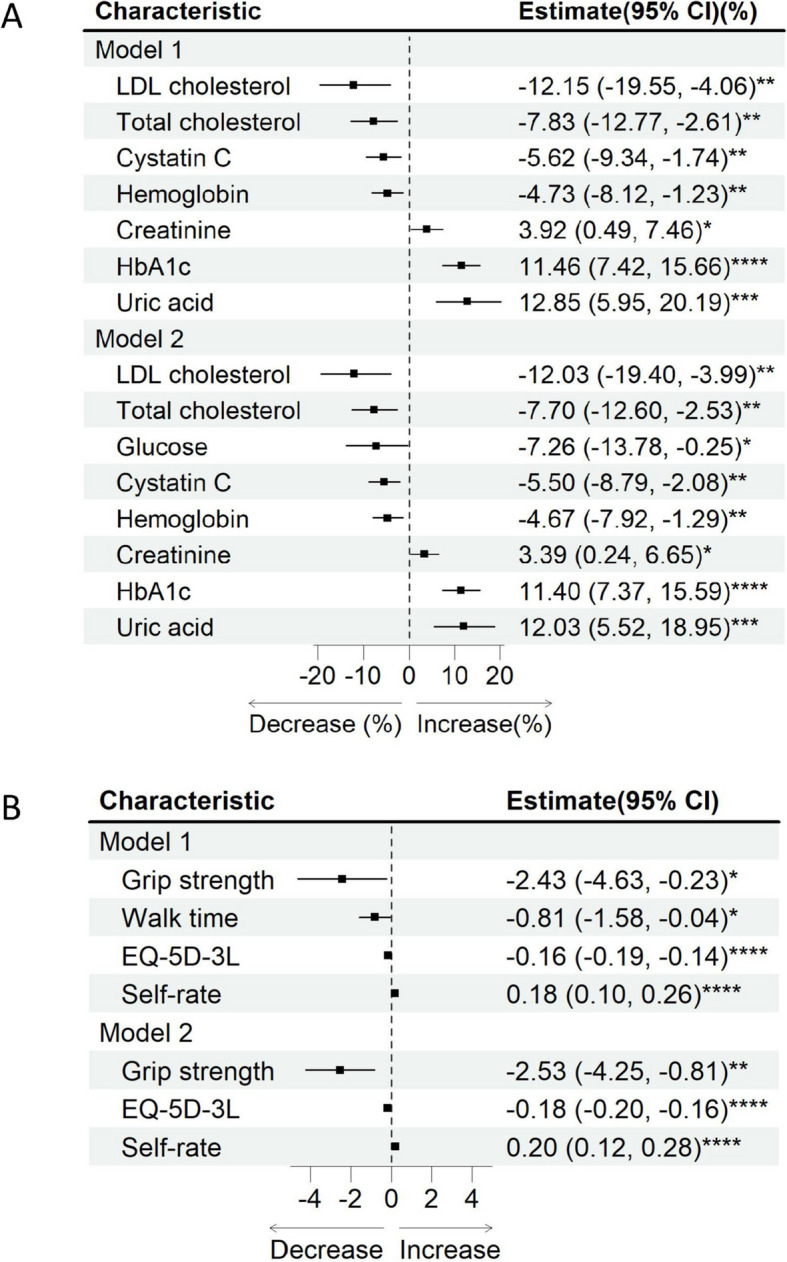
Forest plot of linear regression. Model 1 was not adjusted. Model 2 was adjusted for demographic factors. Only significant outcomes were plotted. Blood analysis outcomes were natural log-transformed and presented as percentage differences (**A**). Other outcomes were not transformed (**B**). *: *p* < 0.05, **: *p* < 0.01, ***: *p* < 0.001, ****: *p* < 0.0001. Abbreviation: EQ-5D-3L: EuroQoL 5-Dimension 3-Level; LDL: low-density lipoprotein; HbA1c: glycosylated hemoglobin, type A1c

Table S-4 presented the results of linear regression analyses examining the relationship between smoking status and all parameters, with all significant outcomes depicted in Fig. 3. A similar trend was observed in Model 1 (M1) and Model 2 (M2), except for several parameters. Notably, the analysis under M2, which accounted for demographic variables, revealed several noteworthy associations. A significant reduction in EQ-5D-3L (β estimate = -0.18) and grip strength (β estimate = -2.53) was observed. However, a significant positive correlation was found between self-rate and smoking cessation (β estimate = 0.20). In the realm of blood analyses, smoking cessation exhibited significant associations with various blood biomarkers after adjusting for demographic factors: 12.03% lower LDL cholesterol, 7.70% lower total cholesterol, 7.26% lower glucose, 5.50% lower cystatin C, 4.67% lower hemoglobin, 3.39% higher creatinine, 11.40% higher HbA1c, and 12.03% higher uric acid.

Considering cessation duration, trend analysis was performed and several significant associations were identified. With each additional year of smoking cessation, self-rate increases by 0.036, while EQ-5D-3L decreases by 0.031. Additionally, glucose decreases by 2.30%, HbA1c increases by 3.29%, uric acid increases by 3.79%, and walk time decreases by 0.474 (Table 4).

**Table 4:** Trend analysis of smoking cessation duration and parameters

	Model 1		Model 2	
	β coefficient (95% CI)	P for trend	β coefficient (95% CI)	P for trend
Self-rate	0.03 (0.01, 0.05)	0.004	0.04 (0.02, 0.06)	0.001
EQ-5D-3L	-0.03 (-0.03, -0.02)	< 0.001	-0.03 (-0.04, -0.03)	< 0.001
Glucose	-2.07 (-4.23, 0.14)	0.066	-2.30 (-4.49, -0.07)	0.044
HbA1c	3.16 (1.93, 4.40)	< 0.001	3.29 (2.05, 4.55)	< 0.001
Uric acid	3.28 (0.73, 5.89)	0.012	3.79 (1.32, 6.33)	0.003
Walk time	-0.45 (-0.79, -0.12)	0.009	-0.47 (-0.84, -0.11)	0.011

Model 1: Not adjusted. Model 2: Adjusted for demographic. Pre: Before cessation. Post: After cessation

All blood analysis data was natural log-transformed, and thus, regression results were presented as percentage differences. Only significate outcomes were displayed

*Abbreviation*: *EQ-5D-3L* EuroQoL 5-Dimension 3-Level, *HbA1c* glycosylated hemoglobin, type A1c

Furthermore, based on the chronic disease classification mentioned above, we divided the respondents into two subgroups: those with smoke-related diseases and those without (Table S-5, Table S-6). However, a similar trend was shown in the two groups and no distinctive outcomes were observed.

Additionally, significant associations were confirmed between smoking behavior and certain parameters. Current smokers demonstrated a worse health status in comparison to non-smokers, which is suitable for prevailing perceptions (Table S-7).

## Discussion

Our discoveries underscored the manifold advantages of abstaining from smoking, consistent with the prevailing scientific consensus regarding the adverse health consequences of tobacco consumption. We successfully quantify the enhancements in health status and blood indicators.

Our findings revealed noteworthy enhancements in specific health markers. The rise in hemoglobin levels can be attributed to the formation of carboxyhemoglobin, a product of the combination of carbon monoxide released from smoke and hemoglobin, leading to tissue hypoxia, heightened erythropoietin secretion, and increased erythropoiesis [33]. Elevated serum cholesterol and LDL levels, previously noted in smokers and considered a risk factor for cardiovascular disease [34], may result from modification of apolipoproteins [35]. Our findings demonstrate an improving trend after smoking cessation. In a prior meta-analysis, smokers were found to have higher HbA1c levels than non-smokers [36], but we found HbA1c increased after cessation, which was similar to other researchers’ findings [37, 38]. It was pointed out that the level of HbA1c would not immediately decrease in the short term after cessation. Furthermore, a decreased glucose level was observed after cessation, possibly linked to nicotine’s stimulation of insulin-antagonizing hormones [39]. Prior studies demonstrated a substantial decrease in cystatin C levels after smoking cessation, possibly indicating a reduction in cardiovascular risks [40]. The outcome of our study supported a recovery in blood parameters after smoking cessation.

Individuals who quit smoking demonstrated a notable decline in EQ-5D-3L scores, indicating a potential initial reduction in the quality of life. In previous studies [41–43], the benefits from smoking cessation have been proved in certain chronic diseases, especially in respiratory system disease. Respondents’ self-rate is often based on daily-life feelings like reduced coughing or easier breathing, which may make them more satisfied with their quality of life. However, the improvement in blood analyses may have less impact on the quality of life. The inverse correlation between smoking cessation and EQ-5D-3L scores necessitates cautious interpretation, possibly also attributable to withdrawal symptoms or lifestyle adjustments, which was proved by a decreased AD coefficient. Smokers may encounter irritability, anxiety, and depression upon abstaining from smoking, which is reliably alleviated by smoking, despite the fact that smoking initially induced these psychological disturbances [44]. Since we combined and analyzed the effects of multiple chronic diseases, the impacts of smoking cessation on different chronic diseases may mask each other. In previous researches, the outcome of smoking cessation is still uncertain. There is still debate about the benefits of smoking cessation for patients with different diseases. In COPD patients [42] and psychiatric problems patients [44], smoking cessation would lead to an improvement in quality of life, however, in cardiovascular disease patients [43, 45, 46] and arthritis patients [47], the benefits are still controversial. Meanwhile, it is worth mentioning that few research focused on older adults as we did. In general, the health status deteriorates as people get old. We must pay attention to the impacts brought about by advanced age.

The regression models provide a nuanced comprehension of the correlation between smoking cessation and health outcomes. Each model was meticulously crafted to sequentially account for potential confounding variables, thus isolating the impact of smoking cessation. M1, which specifically adjusts for smoking cessation status, revealed significant associations that were predominantly consistent across the adjusted models (M2) and the trend analysis, underscoring the robustness of our findings. The consistent results across models, particularly the notable associations observed in blood analyses, underscored the potential advantages of smoking cessation. However, the adverse association with EQ-5D-3L highlights the necessity for supportive interventions to address the immediate challenges quitters face.

The CHARLS dataset serves as a robust foundation for analysis due to its extensive sample size and longitudinal framework. However, it is important to acknowledge limitations such as potential selection bias and the generalizability of our findings to non-Chinese populations. Additionally, there remain other confounders that might not have been controlled for, even though we have corrected for a variety of possible confounding factors. The detailed treatment for chronic diseases was hard to classify. For example, we cannot be sure whether Chinese traditional medicine is suitable for patients. Besides, the use of self-made cigarettes by many individuals in rural areas presented difficulties in quantifying smoking amounts and detailing smoking behaviors. Moreover, the absence of access to medical records in the CHARLS dataset meant that respondents relied on memory to report physician-diagnosed diseases, potentially introducing recall bias. Finally, the cross-sectional nature of the data limits our ability to establish causality. Moving forward, it is imperative to conduct future longitudinal studies with larger sample sizes and extended follow-up periods to validate these findings and explore the temporal dynamics of smoking cessation's effects on health outcomes more comprehensively.

These findings advocate for the integration of smoking cessation interventions into routine care for older adults with chronic diseases, suggesting significant health benefits that warrant further exploration in clinical settings. Future studies should examine the specific effects of smoking cessation on various chronic disease populations and investigate its long-term impacts on healthcare utilization and costs.

## Conclusion

The study's findings underscore the significant health benefits of smoking cessation among older Chinese adults with chronic diseases. Despite an initial decline in perceived quality of life, as indicated by EQ-5D-3L, long-term health markers demonstrate substantial improvements. The favorable trends in blood parameters highlight the physiological advantages of abstaining from smoking. These results reinforce the critical need to incorporate smoking cessation assistance into healthcare protocols, especially for elderly individuals dealing with chronic conditions. The study calls for additional research to investigate the precise effects of smoking cessation on different chronic disease populations and the factors driving smokers to quit.

## Supplementary Information

Supplementary Material 1.

## Data Availability

The data used in our study comes from China Health and Retirement Longitudinal Study (CHARLS), a public database. Researchers who want to use these data can visit http://charls.pku.edu.cn/.

## Abbreviations

COPD: Chronic obstructive pulmonary disease
FEV1%: Forced expiratory volume in one second percentage
FVC: Forced vital capacity
6-MWT: 6-Minute walk test
CHARLS: China Health and Retirement Longitudinal Study
EQ-5D-3L: EuroQoL 5-Dimension 3-Level
WBC: White blood cell count
MCV: Mean corpuscular volume
CRP: C-reactive protein
HbA1c: Glycosylated hemoglobin, type A1c
HDL: High-density lipoprotein
LDL: Low-density lipoprotein
BUN: Blood urea nitrogen
BMI: Body mass index
HRQOL: Health-related quality of life
MO: Mobility
SC: Self-care
UA: Usual activities
PD: Pain/discomfort
AD: Anxiety/depression
CESD-10: Center for Epidemiologic Studies Depression Scale-10

## References

[1] Doll R, Hill AB. Smoking and carcinoma of the lung. Br Med J. 1950;2(4682):739–48. 10.1136/bmj.2.4682.739. 14772469 10.1136/bmj.2.4682.739 PMC2038856

[2] Boyle P. The hazards of passive – and active – smoking. N Engl J Med. 1993;328(23):1708–9. 10.1056/NEJM199306103282311. 8487831 10.1056/NEJM199306103282311

[3] Cicco MED, Ragazzo V, Jacinto T. Mortality in relation to smoking: the British doctors study. Breathe. 2016;12(3):275–6. 10.1183/20734735.013416. 28210302 10.1183/20734735.013416 PMC5298160

[4] Prochaska JJ, Das S, Young-Wolff KC. Smoking, mental illness, and public health. Annu Rev Public Health. 2017;38(Volume 38, 2017):165–85. 10.1146/annurev-publhealth-031816-044618. 27992725 10.1146/annurev-publhealth-031816-044618 PMC5788573

[5] Slattery ML, Robison LM, Schuman KL, et al. Cigarette smoking and exposure to passive smoke are risk factors for cervical cancer. JAMA. 1989;261(11):1593–8. 10.1001/jama.1989.03420110069026. 2918652

[6] Bowman TS, Gaziano JM, Buring JE, Sesso HD. A prospective study of cigarette smoking and risk of incident hypertension in women. J Am Coll Cardiol. 2007;50(21):2085–92. 10.1016/j.jacc.2007.08.017. 18021879 10.1016/j.jacc.2007.08.017

[7] Niskanen L, Laaksonen DE, Nyyssönen K, et al. Inflammation, abdominal obesity, and smoking as predictors of hypertension. Hypertension. 2004;44(6):859–65. 10.1161/01.HYP.0000146691.51307.84. 15492131 10.1161/01.HYP.0000146691.51307.84

[8] Wannamethee SG, Shaper AG, Whincup PH, Walker M. Smoking cessation and the risk of stroke in middle-aged men. JAMA. 1995;274(2):155–60. 10.1001/jama.1995.03530020073035. 7596004

[9] Eder Waltraud, Ege Markus J., von Mutius Erika. The Asthma Epidemic. N Engl J Med. 2006;355(21):2226–2235. 10.1056/NEJMra054308 10.1056/NEJMra054308 17124020

[10] Yusuf S, Hawken S, Ôunpuu S, et al. Effect of potentially modifiable risk factors associated with myocardial infarction in 52 countries (the INTERHEART study): case-control study. Lancet. 2004;364(9438):937–52. 10.1016/S0140-6736(04)17018-9. 15364185 10.1016/S0140-6736(04)17018-9

[11] Rigotti NA, Kruse GR, Livingstone-Banks J, Hartmann-Boyce J. Treatment of tobacco smoking: a review. JAMA. 2022;327(6):566. 10.1001/jama.2022.0395. 35133411 10.1001/jama.2022.0395

[12] Tønnesen P. Smoking cessation and COPD. Eur Respir Rev. 2013;22(127):37–43. 10.1183/09059180.00007212. 23457163 10.1183/09059180.00007212 PMC9487432

[13] Han M, Fu Y, Ji Q, Deng X, Fang X. The effectiveness of theory-based smoking cessation interventions in patients with chronic obstructive pulmonary disease: a meta-analysis. BMC Public Health. 2023;23(1):1510. 10.1186/s12889-023-16441-w. 37559043 10.1186/s12889-023-16441-w PMC10410903

[14] Ho LLK, Li WHC, Cheung AT. Helping patients with chronic diseases quit smoking by understanding their risk perception, behaviour, and smoking-related attitudes. PLoS ONE. 2023;18(4):e0284690. 10.1371/journal.pone.0284690. 37079577 10.1371/journal.pone.0284690 PMC10118143

[15] Wang Z, Qiu Y, Ji X, Dong L. Effects of smoking cessation on individuals with COPD: a systematic review and meta-analysis. Front Public Health. 2024;12:1433269. 10.3389/fpubh.2024.1433269. 39722704 10.3389/fpubh.2024.1433269 PMC11668769

[16] Pan B, Jin X, Jun L, Qiu S, Zheng Q, Pan M. The relationship between smoking and stroke: a meta-analysis. Medicine (Baltimore). 2019;98(12):e14872. 10.1097/MD.0000000000014872. 30896633 10.1097/MD.0000000000014872 PMC6708836

[17] Thomson NC, Polosa R, Sin DD. Cigarette smoking and asthma. J Allergy Clin Immunol Pract. 2022;10(11):2783–97. 10.1016/j.jaip.2022.04.034. 35533997 10.1016/j.jaip.2022.04.034

[18] Rallidis LS, Xenogiannis I, Brilakis ES, Bhatt DL. Causes, angiographic characteristics, and management of premature myocardial infarction: JACC state-of-the-art review. J Am Coll Cardiol. 2022;79(24):2431–49. 10.1016/j.jacc.2022.04.015. 35710195 10.1016/j.jacc.2022.04.015

[19] Wang M, Luo X, Xu S, et al. Trends in smoking prevalence and implication for chronic diseases in China: serial national cross-sectional surveys from 2003 to 2013. Lancet Respir Med. 2019;7(1):35–45. 10.1016/S2213-2600(18)30432-6. 30482646 10.1016/S2213-2600(18)30432-6

[20] Tobacco control in China. Lancet Public Health. 2023;8(12):e1006-e1015. 10.1016/S2468-2667(23)00242-6 10.1016/S2468-2667(23)00242-6 38000880

[21] Jing Z, Li J, Wang Y, et al. Association of smoking status and health-related quality of life: difference among young, middle-aged, and older adults in Shandong. China Qual Life Res. 2021;30(2):521–30. 10.1007/s11136-020-02645-9. 32989682 10.1007/s11136-020-02645-9

[22] Zhao Y, Hu Y, Smith JP, Strauss J, Yang G. Cohort profile: the China Health and Retirement Longitudinal Study (CHARLS). Int J Epidemiol. 2014;43(1):61–8. 10.1093/ije/dys203. 23243115 10.1093/ije/dys203 PMC3937970

[23] von Elm E, Altman DG, Egger M, et al. The Strengthening the Reporting of Observational Studies in Epidemiology (STROBE) statement: guidelines for reporting observational studies. Ann Intern Med. 2007;147(8):573–7. 10.7326/0003-4819-147-8-200710160-00010. 17938396 10.7326/0003-4819-147-8-200710160-00010

[24] Cho ER, Brill IK, Gram IT, Brown PE, Jha P. Smoking cessation and short- and longer-term mortality. NEJM Evid. 2024;3(3):EVIDoa2300272. 10.1056/EVIDoa2300272. 38329816 10.1056/EVIDoa2300272

[25] Alharthi SSY, Natto ZS, Midle JB, Gyurko R, O’Neill R, Steffensen B. Association between time since quitting smoking and periodontitis in former smokers in the National Health and Nutrition Examination Surveys (NHANES) 2009 to 2012. J Periodontol. 2019;90(1):16–25. 10.1002/JPER.18-0183. 30102767 10.1002/JPER.18-0183

[26] Chen X, Crimmins E, Hu P (Perry), et al. Venous Blood-Based Biomarkers in the China Health and Retirement Longitudinal Study: Rationale, Design, and Results From the 2015 Wave. Am J Epidemiol. 2019;188(11):1871–1877. 10.1093/aje/kwz170 10.1093/aje/kwz170 PMC6825825 31364691

[27] Brooks R. EuroQol: the current state of play. Health Policy. 1996;37(1):53–72. 10.1016/0168-8510(96)00822-6. 10158943 10.1016/0168-8510(96)00822-6

[28] Wang S, Shen C, Yang S. Analysis of health-related quality of life in elderly patients with stroke complicated by hypertension in China using the EQ-5D-3L Scale. J Multidiscip Healthc. 2024;17:1981–97. 10.2147/JMDH.S459629. 38706498 10.2147/JMDH.S459629 PMC11069374

[29] Lu S, Wu Y, Mao Z, Liang X. Association of formal and informal social support with health-related quality of life among Chinese rural elders. Int J Environ Res Public Health. 2020;17(4):1351. 10.3390/ijerph17041351. 32093116 10.3390/ijerph17041351 PMC7068316

[30] Liu GG, Wu H, Li M, Gao C, Luo N. Chinese time trade-off values for EQ-5D health states. Value Health. 2014;17(5):597–604. 10.1016/j.jval.2014.05.007. 25128053 10.1016/j.jval.2014.05.007

[31] Boey KW. Cross-validation of a short form of the CES-D in Chinese elderly. Int J Geriatr Psychiatry. 1999;14(8):608–17. 10.1002/(SICI)1099-1166(199908)14:8%3c608::AID-GPS991%3e3.0.CO;2-Z. 10489651 10.1002/(sici)1099-1166(199908)14:8<608::aid-gps991>3.0.co;2-z

[32] Cheng ST, Chan ACM. The center for epidemiologic studies depression scale in older Chinese: thresholds for long and short forms. Int J Geriatr Psychiatry. 2005;20(5):465–70. 10.1002/gps.1314. 15852439 10.1002/gps.1314

[33] Shakiba E, Moradinazar M, Rahimi Z, Najafi F, Pasdar Y, Kohsari M. Tobacco smoking and blood parameters in the kurdish population of Iran. BMC Cardiovasc Disord. 2023;23(1):401. 10.1186/s12872-023-03433-2. 37580672 10.1186/s12872-023-03433-2 PMC10426089

[34] Ambrose JA, Barua RS. The pathophysiology of cigarette smoking and cardiovascular disease: An update. J Am Coll Cardiol. 2004;43(10):1731–7. 10.1016/j.jacc.2003.12.047. 15145091 10.1016/j.jacc.2003.12.047

[35] Zong C, Song G, Yao S, et al. Cigarette smoke exposure impairs reverse cholesterol transport which can be minimized by treatment of hydrogen-saturated saline. Lipids Health Dis. 2015;14(1):159. 10.1186/s12944-015-0160-9. 26634341 10.1186/s12944-015-0160-9 PMC4668613

[36] Soulimane S, Simon D, Herman WH, et al. HbA1c, fasting and 2 h plasma glucose in current, ex- and never-smokers: a meta-analysis. Diabetologia. 2014;57(1):30–9. 10.1007/s00125-013-3058-y. 24065153 10.1007/s00125-013-3058-y PMC4240946

[37] Iino K, Iwase M, Tsutsu N, Iida M. Smoking cessation and glycaemic control in type 2 diabetic patients. Diabetes Obes Metab. 2004;6(3):181–6. 10.1111/j.1462-8902.2004.00329.x. 15056125 10.1111/j.1462-8902.2004.00329.x

[38] Lycett D, Nichols L, Ryan R, et al. The association between smoking cessation and glycaemic control in patients with type 2 diabetes: a THIN database cohort study. Lancet Diabetes Endocrinol. 2015;3(6):423–30. 10.1016/S2213-8587(15)00082-0. 25935880 10.1016/S2213-8587(15)00082-0

[39] Eliasson B. Cigarette smoking and diabetes. Prog Cardiovasc Dis. 2003;45(5):405–13. 10.1053/pcad.2003.00103. 12704597 10.1053/pcad.2003.00103

[40] Funamoto M, Shimizu K, Sunagawa Y, et al. Serum Cystatin C, a sensitive marker of renal function and cardiovascular disease. Decreases After Smoking Cessation Circ Rep. 2019;1(12):623–7. 10.1253/circrep.CR-19-0052. 33693109 10.1253/circrep.CR-19-0052 PMC7897701

[41] Anthonisen NR. Smoking, lung function, and mortality. Thorax. 2000;55(9):729–30. 10.1136/thorax.55.9.729. 10950888 10.1136/thorax.55.9.729 PMC1745844

[42] Han M, Fu Y, Ji Q, Deng X, Fang X. The effectiveness of theory-based smoking cessation interventions in patients with chronic obstructive pulmonary disease: a meta-analysis. BMC Public Health. 2023;23:1510. 10.1186/s12889-023-16441-w. 37559043 10.1186/s12889-023-16441-w PMC10410903

[43] Buchanan DM, Arnold SV, Gosch KL, et al. The association of smoking status with angina and health-related quality of life after acute myocardial infarction. Circ Cardiovasc Qual Outcomes. 2015;8(5):493–500. 10.1161/CIRCOUTCOMES.114.001545. 26307130 10.1161/CIRCOUTCOMES.114.001545 PMC4703446

[44] Taylor G, McNeill A, Girling A, Farley A, Lindson-Hawley N, Aveyard P. Change in mental health after smoking cessation: systematic review and meta-analysis. BMJ. 2014;348:g1151. 10.1136/bmj.g1151. 24524926 10.1136/bmj.g1151 PMC3923980

[45] Quist-Paulsen P, Bakke PS, Gallefoss F. Does smoking cessation improve quality of life in patients with coronary heart disease? Scand Cardiovasc J SCJ. 2006;40(1):11–6. 10.1080/14017430500384855. 16448992 10.1080/14017430500384855

[46] Wu AD, Lindson N, Hartmann-Boyce J, et al. Smoking cessation for secondary prevention of cardiovascular disease. Cochrane Database Syst Rev. 2022;2022(8):CD014936. 10.1002/14651858.CD014936.pub2. 10.1002/14651858.CD014936.pub2 PMC9358996 35938889

[47] Alfredsson L, Klareskog L, Hedström AK. Influence of smoking on disease activity and quality of life in patients with rheumatoid arthritis: results from a swedish case-control study with longitudinal follow-up. Arthritis Care Res. 2023;75(6):1269–77. 10.1002/acr.25026. 10.1002/acr.25026 36149365

