# Supplementary material for: Smoking cessation improves health status of patients with chronic diseases: evidence from a longitudinal study of older adults in China
Source: BMC Public Health. 2025 Mar 11;25:957. doi: 10.1186/s12889-025-22203-7 (PMC11895389; doi:10.1186/s12889-025-22203-7)
Supplement: Supplementary file 1 — Supplementary Material 1. [file 12889_2025_22203_MOESM1_ESM.pdf]

Table S-1 Units for blood analysis and physical measure outcomes

Parameters	Units
**Blood analyses**	
WBC	10^9/L
Hemoglobin	g/dl
Hematocrit	%
MCV	fL
Platelet	10^9/L
BUN	mg/dL
Creatinine	mg/dL
Total cholesterol	mg/dL
Triglycerides	mg/dL
HDL cholesterol	mg/dL
LDL cholesterol	mg/dL
CRP	mg/L
Glucose	mg/dL
HbA1c	%
Uric acid	mg/dL
Cystatin C	mg/L
**Physical measures**	
Walk time	sec
Systolic pressure	mmHg
Diastolic pressure	mmHg
Pulse	/min
Grip strength	kg
BMI	kg/m^2
Lung function	L/min
Balance	-
Chair 5 sec	sec
Chair 5 num	-

Abbreviation: WBC: white blood cell count; MCV: platelet count, mean corpuscular volume; BUN: blood urea nitrogen; HDL: high-density lipoprotein; LDL: low-density lipoprotein; CRP: C-reactive protein; HbA1c: glycosylated hemoglobin, type A1c; BMI: body mass index.

Table S-2 The EQ-5D-3L scale of Chinese residents

Variable	Level Description	Coefficient
C	Constant if at least one dimension has moderate or extreme problems (level 2 or 3)	0.067
N3	Additional constant if at least one dimension has extreme problems (level 3)	0.016
Mobility (MO)		
MO1	No problems with mobility	0.000
MO2	Moderate problems with mobility	0.101
MO3	Extreme problems with mobility	0.275
Self-Care (SC)		
SC1	No problems with performing self-care tasks	0.000
SC2	Moderate problems with performing self-care tasks	0.103
SC3	Extreme problems with performing self-care tasks	0.239
Usual Activities (UA)		
UA1	No problems with performing usual activities	0.000
UA2	Moderate problems with performing usual activities	0.086
UA3	Extreme problems with performing usual activities	0.217
Pain/Discomfort (PD)		
PD1	No pain or discomfort	0.000
PD2	Moderate pain or discomfort	0.110
PD3	Extreme pain or discomfort	0.232
Anxiety/Depression (AD)		
AD1	No feelings of anxiety or depression	0.000
AD2	Moderate feelings of anxiety or depression	0.074
AD3	Extreme feelings of anxiety or depression	0.172

Formula: U = 1 - C - N3 - (MO + SC + UA + PD + AD).

Table S-3 The linear regression between smoking cessation and five dimensions in EQ-5D-3L

Characteristic	Estimate (95% CI)
Model 1	
Mobility (MO)	0.001 (-0.008, 0.009)
Self-care (SC)	0.014 (0.007, 0.021) ****
Usual activities (UA)	0.016 (0.009, 0.022) ****
Pain/discomfort (PD)	0.013 (0.004, 0.023) **
Anxiety/depression (AD)	0.114 (0.107, 0.121) ****
Model 2	
Mobility (MO)	0.004 (-0.005, 0.013)
Self-care (SC)	0.017 (0.010, 0.024) ****
Usual activities (UA)	0.018 (0.012, 0.025) ****
Pain/discomfort (PD)	0.015 (0.006, 0.025) **
Anxiety/depression (AD)	0.117 (0.110, 0.124) ****

*: $p < 0.05$, **: $p < 0.01$, ***: $p < 0.001$, ****: $p < 0.0001$.

Model 1: Not adjusted. Model 2: Adjusted for demographic.

Table S-4 The linear regression between smoking cessation and parameters

	Model 1		Model 2	
	Pre	Post<br>β coefficient (95% CI)	Pre	Post<br>β coefficient (95% CI)
Self-rate	Reference	0.18 (0.10-0.26) ****	Reference	0.20 (0.12-0.28) ****
EQ-5D-3L		-0.16 (-0.19--0.14) ****		-0.18 (-0.20--0.16) ****
**Blood analyses**[1]				
WBC	Reference	-1.63 (-8.06-5.24)	Reference	-1.75 (-8.16-5.12)
Hemoglobin		-4.73 (-8.12--1.23) **		-4.67 (-7.92--1.29) **
Hematocrit		-0.99 (-4.58-2.72)		-1.04 (-4.52-2.56)
MCV		-0.13 (-2.74-2.54)		-0.22 (-2.80-2.44)
Platelet		0.5 (-9.30-11.35)		1.03 (-8.75-11.86)
BUN		-1.35 (-8.31-6.13)		-1.92 (-8.68-5.33)
Creatinine		3.92 (0.49-7.46) *		3.39 (0.24-6.65) *
Total cholesterol		-7.83 (-12.77--2.61) **		-7.70 (-12.60--2.53) **
Triglycerides		7.53 (-7.76-25.37)		8.12 (-7.10-25.83)
HDL cholesterol		-0.49 (-7.63-7.21)		-0.50 (-7.57-7.10)
LDL cholesterol		-12.15 (-19.55--4.06) **		-12.03 (-19.40--3.99) **
CRP		3.36 (-15.70-26.72)		2.33 (-16.42-25.27)
Glucose		-7.00 (-13.56-0.07)		-7.26 (-13.78--0.25) *
HbA1c		11.46 (7.42-15.66) ****		11.4 (7.37-15.59) ****
Uric acid		12.85 (5.95-20.19) ***		12.03 (5.52-18.95) ***
Cystatin C		-5.62 (-9.34--1.74) **		-5.5 (-8.79--2.08) **
**Physical measures**				
Walk time	Reference	-0.81 (-1.58--0.04) *	Reference	-0.57 (-1.28-0.14)
Systolic pressure		1.21 (-3.68-6.10)		1.48 (-3.22-6.18)
Diastolic pressure		1.80 (-0.98-4.58)		1.73 (-1.04-4.49)
Pulse		-0.23 (-2.59-2.12)		-0.26 (-2.62-2.09)
Grip strength		-2.43 (-4.63--0.23) *		-2.53 (-4.25--0.81) **
BMI		0.38 (-3.28-4.04)		0.30 (-3.36-3.95)
Lung function		-4.28 (-31.58-23.02)		-6.56 (-29.52-16.4)
Balance		0.03 (-0.06-0.13)		0.03 (-0.06-0.12)
Chair 5 sec		-0.45 (-1.49-0.59)		-0.42 (-1.36-0.53)
Chair 5 num		0.00 (-0.04-0.04)		0.00 (-0.04-0.04)

*: $p < 0.05$, **: $p < 0.01$, ***: $p < 0.001$, ****: $p < 0.0001$.

Model 1: Not adjusted. Model 2: Adjusted for demographic. Pre: Before cessation. Post: After cessation.

1 All blood analysis data was natural log-transformed, and thus, regression results were presented as percentage differences.

Abbreviation: EQ-5D-3L: EuroQoL 5-Dimension 3-Level; WBC: white blood cell count; MCV: platelet count, mean corpuscular volume; BUN: blood urea nitrogen; HDL: high-density lipoprotein; LDL: low-density lipoprotein; CRP: C-reactive protein; HbA1c: glycosylated hemoglobin, type A1c; BMI: body mass index.

Table S-5 The linear regression between smoking cessation and parameters (With smoke-related diseases)

	Model 1		Model 2	
	Pre	Post<br>β coefficient (95% CI)	Pre	Post<br>β coefficient (95% CI)
Self-rate	Reference	0.21 (0.10-0.31) ***	Reference	0.22 (0.11-0.32) ****
EQ-5D-3L		-0.18 (-0.21--0.14) ****		-0.19 (-0.23--0.16) ****
**Blood analyses**[1]				
WBC	Reference	-0.13 (-8.44-8.93)	Reference	-0.21 (-8.50-8.83)
Hemoglobin		-6.09 (-10.41--1.56) **		-6.18 (-10.32--1.84) **
Hematocrit		-2.00 (-6.56-2.77)		-2.18 (-6.59-2.43)
MCV		0.57 (-2.68-3.93)		0.51 (-2.72-3.85)
Platelet		8.23 (-5.08-23.41)		8.74 (-4.53-23.84)
BUN		1.22 (-7.98-11.35)		0.73 (-8.23-10.55)
Creatinine		3.48 (-1.06-8.22)		2.99 (-1.19-7.34)
Total cholesterol		-8.57 (-14.81--1.86) *		-8.44 (-14.63--1.80) *
Triglycerides		-9.02 (-25.42-10.97)		-8.45 (-24.76-11.38)
HDL cholesterol		5.00 (-4.70-15.69)		4.87 (-4.70-15.40)
LDL cholesterol		-11.62 (-21.26--0.78) *		-11.52 (-21.13--0.75) *
CRP		-3.44 (-26.40-26.69)		-4.04 (-26.76-25.73)
Glucose		-12.88 (-20.86--4.09) **		-12.98 (-20.94--4.22) **
HbA1c		11.12 (5.91-16.58) ****		11.12 (5.93-16.58) ****
Uric acid		10.76 (1.87-20.43) *		10.00 (1.60-19.10) *
Cystatin C		-6.78 (-11.77--1.52) *		-6.23 (-10.72--1.51) *
**Physical measures**				
Walk time	Reference	-0.83 (-1.84-0.18)	Reference	-0.55 (-1.49-0.39)
Systolic pressure		-1.47 (-7.84-4.89)		-1.04 (-7.30-5.21)
Diastolic pressure		1.53 (-2.14-5.19)		1.22 (-2.37-4.8)
Pulse		0.14 (-3.02-3.31)		0.13 (-3.04-3.29)
Grip strength		-2.48 (-5.36-0.41)		-3.09 (-5.35--0.84) **
BMI		0.22 (-5.79-6.23)		0.09 (-5.91-6.08)
Lung function		10.38 (-25.56-46.32)		2.08 (-28.64-32.80)
Balance		0.06 (-0.07-0.19)		0.05 (-0.07-0.18)
Chair 5 sec		-0.73 (-2.16-0.71)		-0.60 (-1.92-0.73)
Chair 5 num		0.00 (-0.05-0.05)		0.00 (-0.05-0.05)

*: $p < 0.05$, **: $p < 0.01$, ***: $p < 0.001$, ****: $p < 0.0001$.

Model 1: Not adjusted. Model 2: Adjusted for demographic. Pre: Before cessation. Post: After cessation.

1 All blood analysis data was natural log-transformed, and thus, regression results were presented as percentage differences.

Abbreviation: EQ-5D-3L: EuroQoL 5-Dimension 3-Level; WBC: white blood cell count; MCV: platelet count, mean corpuscular volume; BUN: blood urea nitrogen; HDL: high-density lipoprotein; LDL: low-density lipoprotein; CRP: C-reactive protein; HbA1c: glycosylated hemoglobin, type A1c; BMI: body mass index.

Table S-6 The linear regression between smoking cessation and parameters (Without smoke-related diseases)

	Model 1		Model 2	
	Pre	Post β coefficient (95% CI)	Pre	Post β coefficient (95% CI)
Self-rate	Reference	0.15 (0.03-0.27) *	Reference	0.20 (0.12-0.28) ****
EQ-5D-3L		-0.14 (-0.17--0.11) ****		-0.18 (-0.20--0.16) ****
**Blood analyses**[1]				
WBC	Reference	-1.63 (-8.06-5.24)	Reference	-4.05 (-13.8-6.8)
Hemoglobin		-4.73 (-8.12--1.23) **		-2.01 (-7.16-3.42)
Hematocrit		-0.99 (-4.58-2.72)		0.98 (-4.56-6.83)
MCV		-0.13 (-2.74-2.54)		-1.03 (-5.27-3.4)
Platelet		0.50 (-9.30-11.35)		-9.63 (-23.27-6.43)
BUN		-1.35 (-8.31-6.13)		-5.40 (-15.38-5.75)
Creatinine		3.92 (0.49-7.46) *		4.34 (-0.39-9.30)
Total cholesterol		-7.83 (-12.77--2.61) **		-6.68 (-14.44-1.79)
Triglycerides		7.53 (-7.76-25.37)		38.97 (9.77-75.95) **
HDL cholesterol		-0.49 (-7.63-7.21)		-8.02 (-17.96-3.13)
LDL cholesterol		-12.15 (-19.55--4.06) **		-13.19 (-24.14--0.65) *
CRP		3.36 (-15.7-26.72)		12.20 (-16.89-51.48)
Glucose		-7.00 (-13.56-0.07)		1.76 (-9.02-13.83)
HbA1c		11.46 (7.42-15.66) ****		11.99 (5.70-18.65) ***
Uric acid		12.85 (5.95-20.19) ***		15.63 (5.73-26.46) **
Cystatin C		-5.62 (-9.34--1.74) **		-4.31 (-8.94-0.55)
**Physical measures**				
Walk time	Reference	-0.77 (-1.86-0.32)	Reference	-0.57 (-1.28-0.14)
Systolic pressure		5.15 (-1.25-11.56)		1.48 (-3.22-6.18)
Diastolic pressure		2.01 (-1.93-5.96)		1.73 (-1.04-4.49)
Pulse		-1.00 (-4.48-2.48)		-0.26 (-2.62-2.09)
Grip strength		-2.18 (-5.53-1.18)		-2.53 (-4.25--0.81) **
BMI		0.57 (-1.09-2.24)		0.30 (-3.36-3.95)
Lung function		-21.63 (-61.46-18.21)		-6.56 (-29.52-16.4)
Balance		-0.01 (-0.15-0.13)		0.03 (-0.06-0.12)
Chair 5 sec		-0.07 (-1.48-1.35)		-0.42 (-1.36-0.53)
Chair 5 num		0.00 (-0.04-0.04)		0.00 (-0.04-0.04)

*: $p < 0.05$, **: $p < 0.01$, ***: $p < 0.001$, ****: $p < 0.0001$.

Model 1: Not adjusted. Model 2: Adjusted for demographic. Pre: Before cessation. Post: After cessation.

1 All blood analysis data was natural log-transformed, and thus, regression results were presented as percentage differences.

Abbreviation: EQ-5D-3L: EuroQoL 5-Dimension 3-Level; WBC: white blood cell count; MCV: platelet count, mean corpuscular volume; BUN: blood urea nitrogen; HDL: high-density lipoprotein; LDL: low-density lipoprotein; CRP: C-reactive protein; HbA1c: glycosylated hemoglobin, type A1c; BMI: body mass index.

Table S-7 The linear regression between smoking behavior and parameters

	Model 1		Model 2	
	Pre	Never<br>β coefficient (95% CI)	Pre	Never<br>β coefficient (95% CI)
Self-rate	Reference	0.19 (0.13-0.26) ****	Reference	0.13 (0.05-0.20) ***
EQ-5D-3L		-0.13 (-0.15--0.11) ****		-0.11 (-0.13--0.09) ****
**Blood analyses**[1]				
WBC	Reference	-3.03 (-7.69-1.87)	Reference	-2.05 (-6.89-3.04)
Hemoglobin		-9.43 (-11.78--7.01) ****		-3.03 (-5.53--0.47) *
Hematocrit		-7.75 (-10.19--5.24) ****		-1.86 (-4.45-0.80)
MCV		-2.37 (-4.23--0.47) *		-0.70 (-2.64-1.28)
Platelet		3.00 (-4.42-11.00)		-4.30 (-11.34-3.31)
BUN		-8.65 (-13.42--3.63) ***		-2.85 (-7.95-2.52)
Creatinine		-7.11 (-9.37--4.80) ****		1.11 (-1.23-3.50)
Total cholesterol		2.25 (-1.79-6.47)		-3.33 (-7.22-0.73)
Triglycerides		7.88 (-3.59-20.73)		-4.74 (-15.04-6.81)
HDL cholesterol		5.42 (-0.18-11.34)		3.39 (-2.20-9.29)
LDL cholesterol		2.89 (-3.54-9.75)		-3.51 (-9.67-3.07)
CRP		-2.53 (-16.05-13.18)		-3.14 (-16.85-12.83)
Glucose		-0.56 (-5.76-4.92)		-1.54 (-6.81-4.03)
HbA1c		6.80 (3.96-9.72) ****		5.48 (2.6-8.44) ***
Uric acid		-8.64 (-12.77--4.32) ***		4.81 (0.18-9.66) *
Cystatin C		-6.02 (-8.93--3.02) ***		-3.32 (-6.03--0.54) *
**Physical measures**				
Walk time	Reference	-0.06 (-0.66-0.54)	Reference	-0.24 (-0.81-0.33)
Systolic pressure		1.64 (-2.05-5.32)		2.05 (-1.61-5.71)
Diastolic pressure		-1.80 (-3.9-0.29)		-1.07 (-3.22-1.08)
Pulse		-0.20 (-1.98-1.57)		-1.05 (-2.89-0.78)
Grip strength		-12.29 (-13.94--10.63) ****		-3.51 (-4.85--2.18) ****
BMI		1.65 (-1.12-4.42)		1.12 (-1.74-3.97)
Lung function		-72.42 (-92.97--51.87) ****		1.11 (-16.74-18.96)
Balance		-0.09 (-0.17--0.02) *		0.01 (-0.06-0.08)
Chair 5 sec		0.96 (0.18-1.74) *		0.12 (-0.62-0.86)
Chair 5 num		-0.01 (-0.03-0.02)		0.00 (-0.02-0.03)

*: $p < 0.05$, **: $p < 0.01$, ***: $p < 0.001$, ****: $p < 0.0001$.

Model 1: Not adjusted. Model 2: Adjusted for demographic. Pre: Before cessation. Never: Non-smokers.

1 All blood analysis data was natural log-transformed, and thus, regression results were presented as percentage differences.

Abbreviation: EQ-5D-3L: EuroQoL 5-Dimension 3-Level; WBC: white blood cell count; MCV: platelet count, mean corpuscular volume; BUN: blood urea nitrogen; HDL: high-density lipoprotein; LDL: low-density lipoprotein; CRP: C-reactive protein; HbA1c: glycosylated hemoglobin, type A1c; BMI: body mass index.
